# Enteric glia as friends and foes of the intestinal epithelial barrier function

**DOI:** 10.3389/fimmu.2024.1394654

**Published:** 2024-05-30

**Authors:** Vedrana Bali, Vladimir Grubišić

**Affiliations:** ^1^ Department of Biomedical Sciences, New York Institute of Technology College of Osteopathic Medicine, Old Westbury, NY, United States; ^2^ Center for Biomedical Innovation, New York Institute of Technology College of Osteopathic Medicine, Old Westbury, NY, United States

**Keywords:** enteric nervous system, enteric glia, neurogastroenterology, epithelial barrier function, inflammation, inflammatory bowel disease (IBD), ulcerative colitis, Crohn’s disease

## 1 Introduction

The luminal surface of the digestive tract is one of the largest surfaces where human body meets environment (1). One of the key functions of the gut lining is selective permeability allowing nutrients to cross the barrier while keeping out the pathogens from entering the system. The gut barrier is composed of microbial biofilms, mucus layer, epithelial cells and their tight junctions, and the resident immune system. Increased permeability of the intestinal barrier, colloquially termed “leaky gut”, has been correlated with several gastrointestinal (GI) diseases and extraintestinal disorders including metabolic, neurodegenerative, neurodevelopmental, and psychiatric diseases (2). Abnormal barrier function was also reported in functional GI disorders such as irritable bowel syndrome (3). Gut epithelial barrier dysfunction contributes to the pathophysiology of inflammatory bowel disease (IBD) (4), a debilitating disorder characterized by bouts of acute intestinal inflammation and clinical remission.

The intestinal epithelial barrier is recognized as a potential therapeutic target (5). It is regulated by the interplay between the epithelial cells, resident immune cells, enteric nervous system (ENS), innervation from the central nervous system (CNS), and vasculature (6–8). In this article, we give our attention to the interaction between the epithelial cells, enteric glia, and immunocytes, and how they contribute to the gut epithelial barrier function. Several recent reviews also addressed this topic (9–14), so here we briefly focus on some advances that were not previously discussed in detail.

## 2 Enteric glia and epithelial cells are in proximity and make direct structural and functional connections

**Figure 1:**
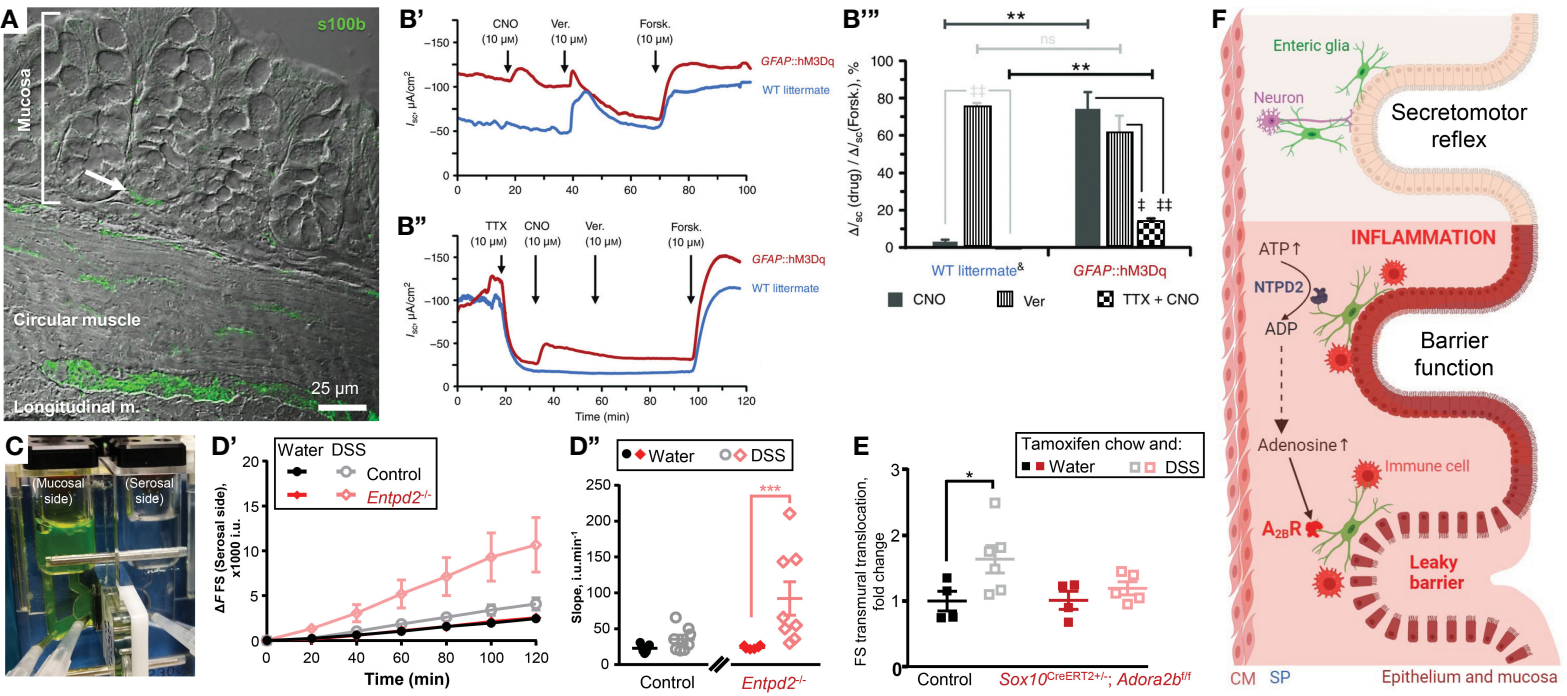
Enteric glia contribute to homeostasis of the gut epithelium in health and disease. **(A, B)** Enteric glia and epithelial cells are in proximity and make functional connections. **(A)** Confocal differential interference contrast image of a transmural section of the mouse colon stained with glial marker s100β (green). Note the s100β positive cells in proximity to the base of the colonic crypt (arrow). **(B)** Glial stimulation emulates neuron‐evoked changes in transepithelial ion movement and is not completely blocked by tetrodotoxin. **(B’)** Representative short-circuit current (*I*_SC_) recordings from distal colon preparations of *GFAP*::hM3Dq mice (red) and their wild‐type (WT) littermates (blue) in response to the hM3Dq agonist clozapine‐N‐oxide (CNO). Neuron depolarization and epithelial cell stimulation were induced by veratridine (Ver.) and the secretagogue forskolin (Forsk), respectively. Drugs were added at times indicated by arrows. Note that WT mice do not respond to CNO. **(B’’)** Representative *I*_SC_ recordings from distal colon preparations pretreated with the voltage‐gated sodium channel inhibitor tetrodotoxin (TTX). Other drugs were added as mentioned above. Note that CNO still elicits response in *GFAP*::hM3Dq mice although veratridine response is completely blocked. **(B’’’)** CNO (dark grey), veratridine (vertical stripes), and TTX‐preincubated CNO (checker board)‐induced responses of WT littermates (left) and *GFAP*::hM3Dq preparations (right) normalized to the individual forskolin responses. Horizontal bars at the top mark comparisons of the same drug treatment between the genotypes. Comparisons of the same drug treatment between the genotypes are marked by horizontal bars at the top. **P < 0.01; ns, not significant, Student’s t test. Comparisons of different drug treatments within the same genotype are marked by angled bars mark. ^‡^P < 0.05; ^‡‡^P < 0.01, analysis of variance (ANOVA) followed by Tukey/Kramer *post hoc* test. ^&^CNO and Ver responses are pooled from vehicle‐treated *GFAP*::hM3Dq mice and CNO‐treated WT littermates. N = 3–6 animals per group. **(C–E)** Glial purinergic signaling has opposite roles in barrier function following acute colitis. **(C)** A photo of a Ussing chamber. A cell impermeable dye fluorescein-sulfonate (FS, 478.32 Da) was added to the mucosal side and the serosal side was sampled every 20 minutes to assess the paracellular permeability. **(D)** Paracellular permeability of the control mice (black and grey) and Ectonucleoside triphosphate diphosphohydrolase-2 knockouts (*Entpd2* ^-/-^, red and pink) two weeks after the dextran sulfate sodium (DSS) treatment (grey, pink) or their healthy littermates (black, red). ***P0.001, two-way ANOVA and Tukey’s *post hoc* test. N = 7=8 mice. **(E)** Glial A_2B_Rs mediate persistent gut barrier dysfunction after inflammation. Normalized rate of transmural FS translocation in the mouse distal colon. *P = 0.032, 2-way ANOVA, Sidak’s multiple comparisons test. N = 4–6 mice per group. **(F)** Schematic depicting glial roles in epithelial functions in health and disease. Enteric glia make structural and functional contacts with the epithelial cells. In health enteric glia regulate secretomotor reflex. During inflammation, enteric glia protect the epithelial barrier via ATP hydrolysis by NTPD2, or exacerbate the barrier dysfunction via adenosine signaling and activation of immunocytes. **(A–E)** were sourced and modified from published literature (15–17). **(F)** was created with BioRender.com. CM, circular muscle; SP, submucosal plexus.

Enteric glia are present in all layers of the gut (**Figure 1A**). Within the intestinal mucosa, glial cells are in proximity to the gut epithelium in humans (18) and mice (Figure A, arrow). 3D electron microscopy revealed cell-to-cell contacts between the mucosal glia and enteroendocrine cells (19), a subtype of the gut epithelial cells. Thus, glial cells are ideally positioned to integrate signals from the ENS and CNS and may directly regulate epithelial functions.

Glia cannot generate action potentials (20), but their activation is encoded by an increase in the cytosolic calcium concentration (21). Chemogenetics is one way to specifically increase glial calcium in intestinal tissue by utilizing Designer Receptors Exclusively Activated by Designer Drugs (DREADD) technology. The selective glial activation evoked ion movement comparable to the direct activation of neurogenic ion transport (15). Glia-driven responses consisted of two components, one sensitive to a neurotoxin tetrodotoxin, and another independent of action potentials (**Figure 1B**). The latter suggests that glial activity regulates ion transport by direct effects on epithelial cells. These findings also place glia as important players in intestinal fluid transport and suggest a new mechanistic target for the treatment of functional diarrheal diseases.

Whether the epithelial cells signal to glia is still an open question. One expectation is that mucosal glia connected to the enteroendocrine cells could regulate the first synapse on the direct route from the gut epithelium to the brain (see below section 5 for more discussion).

## 3 Epithelial barrier function – is this reserved for reactive glia?

The role of enteric glia in regulation of the epithelial barrier is not in agreement, as nicely cataloged in a recent review (14). Here we offer another perspective that could explain this disagreement. We provide evidence for and against the theory that only reactive glia significantly affect the epithelial barrier and that the effect on the barrier is dependent on specific molecular mechanisms.

Enteric glia are heterogeneous and composed of 4 subpopulations based on their location and morphology (22). More recently, single-cell/-nuclei RNA sequencing identified 7 transcriptionally distinct enteric glial cell subtypes (23, 24), but we still do not know the specific biological function of these subpopulations. In addition, enteric glia are plastic, meaning their transcriptome changes in response to injury, infection, and inflammation. This state is known as activated or reactive gliosis and is characterized by upregulation of Gfap expression and downregulation of Plp1 expression (25, 26).

Early data from enteric glial ablation models supported a critical role in barrier function since their ablation produced fulminant jejunoileitis (27). This method of glial ablation included the Gfap promoter-driven expression of thymidine kinase gene of the herpes simplex virus (HSV-Tk) mice that were subsequently treated with ganciclovir. This approach, however, also induces ablation of neighboring cells (28). Glial role in the epithelial barrier function was reinforced by *in vitro* studies which showed that glial mediators such as GDNF, GNSO, and TGFβ have direct effects on epithelial cells (29–31). However, these mediators are not exclusive to glial cells but are also expressed by other cell types in the mucosa, including epithelial and smooth muscle cells, so it is not clear what is the contribution of the glia-produced factors (14). Furthermore, no overt “leaky” gut or intestinal inflammation was observed in scenarios where mucosal glia are absent in germ-free mice (32) or when these cells are ablated using a Plp1 promoter-driven expression of diphtheria toxin (33). The latter also found that ablated Plp1+ cells do not affect the progression of intestinal inflammation induced by dextran sodium sulfate (DSS). Another study used thymidine kinase to ablate GFAP+ glia with reduced ganciclovir dosage and did not see fulminant colitis or a change in the progression of the DSS-induced inflammation (34). This study did not achieve total ablation of GFAP+ cells suggesting that there may be a threshold for glial subpopulation that is required for recovery of normal gut barrier function.

Do enteric glia support the epithelial barrier function *in vivo*? Baghdadi, et al. identified 3 mucosal subgroups: Gfap^High^/Plp1^Low^, Gfap^Low^/Plp1^High^, and Gfap^Mid^/Plp1^Low^ and combined diphtheria toxin-induced ablation of Gfap+ and Plp1+ cells (25). They found that these populations of enteric glia have redundant homeostatic roles, but the GFAP+ subpopulation of enteric glia plays a particularly important role in the mucosa by promoting epithelial regeneration through effects on the self-renewal of intestinal stem cells (25). This study also suggested that Plp1+ cells serve as a reserve pool for Gfap+ cells and gut inflammation induces the transition of Plp1+ cells into Gfap+ cells. The effects of glia on the epithelial barrier also depend on the extracellular environment and cell signaling mechanism. Activation or suppression of glial Ca^2+^ signaling does not change transepithelial conductance or paracellular permeability in healthy intestine (15). However, ablating NTPDase2, an ATP hydrolyze that is predominantly expressed by enteric glia, increases paracellular permeability during acute DSS colitis (16) (**Figures 1C, D**).

Taken together, it looks like the enteric glia in the healthy intestine play redundant roles in barrier integrity, but are important during intestinal inflammation or tissue injury when glia become reactive and increase GFAP expression (35). Of note, at basal state enteric glia express higher levels of GFAP than other cell types that also express GFAP such as astrocytes and Schwann cells, presumably a consequence of cues from the gut microenvironment (36). Upregulation of GFAP expression is a sign of reactive gliosis that changes the phenotype of the glial cells (9). Likewise, most of the *in-vitro* work may be also dealing with the reactive glia because culturing increases GFAP expression, a sign of glial reactivity. Some of the reactivity seen/observed *in vitro* could be controlled by reducing the levels of serum in the culture media (37).

These findings suggest that enteric glia play a more prominent role in barrier regulation during inflammation than in health. Indeed, glia play a critical role in recovery from ischemic injury during early postnatal period (38). It would be interesting to see how young germ-free mice without mucosal glia (32) would behave when challenged by acute intestinal inflammation or ischemic injury. Of note, human mucosal glial cells are present at birth and their homeostasis is independent of microbiota (39).

## 4 Glial purine signaling in acute colitis as a double-edged sword for the barrier function

Purine nucleotides are involved in the regulation of many processes including inflammation. During the initial inflammation phase, there is a surge of ATP that serves as a cell death signal and acts proinflammatory. During intestinal inflammation, ATP causes P2X7-depended death of enteric neurons (40) and P2X2-dependent enteric gliosis (41). Extracellular ATP is degraded by ectonucleotidases nucleoside triphosphate diphosphohydrolases (NTPDases), these enzymes are expressed on the extracellular side of the cell membrane and they hydrolase ATP into ADP and AMP. NTPDase2 is expressed almost exclusively by enteric glia and ablation of the NTPDase2 encoding gene increased gut permeability following chemically-induced colitis (16) (**Figures 1C, D**). In other words, glia-specific extracellular nucleotide phosphohydrolysis by NTPDase2 substantially reduces gut barrier dysfunction during intestinal inflammation. This outcome is relevant for the development of novel ways of preventing or postponing IBD flareups and improving the quality of life during remission. Many patients in clinical remission still suffer from an underlying “leaky gut” and complain of abdominal pain and diarrhea (42). The persistent epithelial barrier dysfunction during the IBD remission also speeds up the advent of new episodes of active IBD (43).

The enzymatic degradation of ATP leads to an increase in adenosine, traditionally considered an anti-inflammatory molecule important for the resolution of inflammation. This increased adenosine concentration activates the exclusive low-affinity adenosine 2B receptors (A_2B_R). However, global knockout animals or the use of the A_2B_R inhibitors resulted in opposing results which suggested a cell type-specific A_2B_R signaling. Indeed, specific deletion of the A_2B_R from the epithelial cells worsened the colitis outcomes (44) while glia-specific A_2B_R ablation ameliorated the inflammation-induced increase in the tissue damage (17) (**Figure 1E**).

In summary, glial purinergic signaling has opposing outcomes in acute inflammation (**Figures 1D, E**). Ablation of the predominantly glial NTPDase2 increased gut permeability following dextran sodium sulfate (DSS) colitis (16) while deleting glial A_2B_Rs prevented the inflammation-induced increase in permeability (17). These observations indicate that enteric glia play complex roles in the intestinal epithelial barrier during inflammation that can result in very different functional outcomes.

The caveat in the comparison of these two studies is that glial A_2B_R ablated mice and their wt controls were treated with tamoxifen and DSS while NTPDase2 null mice and their wt littermates were treated with only DSS. Note that wt controls recovered from DSS after two weeks (**Figure 1D’’**), but tamoxifen-treated wt mice have a prolonged DSS-induced permeability (**Figure 1E**). This indicates that tamoxifen co-administration via chow can exacerbate tissue damage and increase gut dysfunction. Indeed, tamoxifen administration in mice and humans showed altered gastrointestinal motility (45) and tamoxifen-induced intestinal epithelial barrier damage (46), respectively. Therefore, tamoxifen administration should be controlled for the off-target effects of tamoxifen treatment in GI.

## 5 Gut epithelial barrier and the gut-brain axis

As already mentioned above, mucosal glia are in the perfect position to integrate signals from the ENS and CNS innervation. For example, cholinergic signaling in the gut protects the intestinal barrier through activation of enteric glia (47). Enteric glia are critical for the vagal nerve stimulation-induced limitation of intestinal inflammation following injury (48). The lining of the gut communicates with the CNS by direct innervation and via enteroendocrine hormones. Enteroendocrine cells synapse to the vagal afferents (49) and they are in direct contact with enteric glia (19) but we still don’t know the physiological impact of glia-specific signaling. Perhaps glial modulation of enteroendocrine cells or synaptic function between enteroendocrine cells and intrinsic/extrinsic neurons affects the production and/or release of GLP-2, a known enhancer of the epithelial barrier function (50). Another way that the gut can communicate with the brain is through intestinal immunocytes and immune mediators that travel from the GI system into systemic circulation, and reach the brain (51). Enteric glia communicate with intestinal immunocytes such as macrophages, monocytes, lymphocytes, and innate lymphoid cells (26). It would be interesting to investigate glial roles in these modes of communication in the gut-brain-gut axes.

Enteric glia play an important role in several extraintestinal disorders. One of the most common neurodegenerative disorders, Parkinson’s disease, is characterized by GI symptoms occurring at any stage, even preceding the onset of CNS motor dysfunction in a significant number of patients. Enteric glia exhibit changes during the earliest stages of Parkinson disease (52), and they may play a major role in the disease development and progression in the central nervous system through modulation of the intestinal barrier, microbiota, and inflammation (53). Enteric glia also mediate the impaired gut barrier function in psychiatric disorders such as anxiogenic and depressive-like behaviors (54). In addition, enteric glia are the site of entry to the CNS in infections such as prion disease (55).

Just as enteric glia maintain fast bi-directional communication with neurons, which is crucial in the regulation of GI functions, recent studies suggest enteric glia may play a bi-directional role in gut-brain axis as well. Researchers identified a key role that enteric glia have in behavioral changes related to compromised gut epithelial barrier function caused by low-grade inflammation induced by chronic high-fat diet. The authors prevented development of depressive- and anxiety-like behaviors in animals on high-fat diet by disrupting the function of enteric glia (54). Enteric glia have a critical role in mediating the effects of psychological stress on gut function via glia-dependent activation of intestinal macrophages (56). This is an important pathophysiological connection from psychological stress to intestinal inflammation.

## 6 Conclusion

Enteric glia within intestinal mucosa are perfectly positioned to interact with the innervation from the ENS and the CNS, gut immune system, and intestinal epithelial cells. These glial cells have active roles in the regulation of gut reflexes such as secretomotor function (**Figure 1F**). Some roles of enteric glial cells within the healthy intestine seem to be redundant, such as epithelial barrier function. However, in inflamed and/or injured intestines enteric glia have important roles in tissue repair, glia-immunocyte interactions as well as tissue recovery (**Figure 1F**). Future studies are needed to investigate the mechanisms of these interactions.

## Author contributions

VB: Conceptualization, Writing – original draft, Writing – review & editing. VG: Conceptualization, Data curation, Funding acquisition, Visualization, Writing – original draft, Writing – review & editing.
